# S167. ANATOMICAL CONNECTIVITY OF THE VISUOSPATIAL ATTENTIONAL NETWORK IN SCHIZOPHRENIA: A DTI-BASED TRACTOGRAPHY STUDY

**DOI:** 10.1093/schbul/sby018.954

**Published:** 2018-04-01

**Authors:** Leroux Elise, Poirel Nicolas, Sonia Dollfus

**Affiliations:** 1 Service de Psychiatry Chu de Caen; 2 Université Paris Descartes, Normandie Univ, UNICAEN, Institut Universitaire de France (IUF); 3 Centre Hospitalier Universitaire de Caen, Centre Esquirol

## Abstract

**Background:**

In healthy controls (HCs), the visuospatial attentional network consists of fronto-parietal bundles distributed across both hemispheres, but the anatomical organization of this network remains largely unknown in patients with schizophrenia (SZPs). Using diffusion tensor imaging (DTI)-based tractography, we investigated both white matter integrity and the volume of visuospatial attentional pathways in the right and left hemispheres (RH and LH), as well as their structural asymmetry in SZPs and HCs and hypothesized that SZPs would have WM pathway alterations and abnormal structural asymmetry.

**Methods:**

This study included 34 SZPs and 69 HCs. Integrity parameters (fractional anisotropy [FA], radial and mean diffusivity [RD and MD]) and volume were calculated in each fasciculus of this network: the three branches of the superior longitudinal fasciculus (SLFI–III), the inferior longitudinal fasciculus, and the inferior fronto-occipital fasciculus in the RH and LH.

**Results:**

In SLFII and SLFIII, compared to RH and LH values for SZPs, HCs presented increased FA and/or decreased RD/MD in RH and/or LH. Both SZPs and HCs presented increased FA and decreased RD/MD in the RH compared to the LH in the SLFIII, whereas only HCs had this pattern in the SLFII. Volumes did not differ between groups.

**Discussion:**

To our knowledge, this study is the first to describe the structural hemispheric lateralization/organization of the visuospatial attentional network in SZPs. Our main findings are disrupted structural connectivity in the SLFII associated with abnormal anatomical asymmetry in patients, which could be a substrate of attentional deficits.

